# Heat-related urban mortality attributable to human-induced climate change in Europe in summer 2026

**DOI:** 10.1038/s44360-026-00209-8

**Published:** 2026-10-14

**Authors:** Clair Barnes, Pierre Masselot, Di Xi, Archie Rayner, Xinyi Chen, Emmanuel Raju, Malcolm Mistry, Antonio Gasparrini, Friederike Otto, Ana Maria Vicedo-Cabrera, Garyfallos Konstantinoudis

**Affiliations:** 1 Centre for Environmental Policy, Imperial College London, London, UK; 2 Environment & Health Modelling Lab, London School of Hygiene & Tropical Medicine, London, UK; 3 School of Public Health, Imperial College London, London, UK; 4 Department of Public Health, University of Copenhagen, Copenhagen, Denmark; 5 Department of Economics, Ca’ Foscari University of Venice, Venice, Italy; 6 Institute of Social and Preventive Medicine, Universität Bern, Bern, Switzerland; 7 Oeschger Center for Climate Change Research, Universität Bern, Bern, Switzerland; 8 Grantham Institute for Climate Change and the Environment, Imperial College London, London, UK

## Abstract

The summer of 2026 was characterized by a series of extreme heatwaves across Western Europe, with both monthly and annual temperature records broken repeatedly across many countries from May onwards. Several government agencies took the unprecedented step of issuing near-real-time estimates of the mortality associated with the most intense heat events, reflecting the severity of the threat to public health. Here we combine probabilistic climate attribution with city- and age-specific temperature– mortality relationships to estimate heat-related mortality in 854 European cities during the summer months (June–August) and compare with counterfactual climate conditions representing a world without human-caused warming. We estimate 50,391 heat-related excess deaths across the 854 cities during the summer, with around two-thirds of these attributable to anthropogenic climate change. These results highlight the threat from human-caused warming and the need for rapid emissions reductions alongside preparation and adaptation measures.

The summer of 2026 has been confirmed as the third hottest on record over Europe and the hottest ever for western Europe (https://climate.copernicus.eu/copernicus-august-was-worlds-joint-hottest-month-record-pushing-global-temperatures-back-above). The season was characterized by a series of heatwaves, beginning in late May and persisting until the end of August, across western Europe and large parts of central, eastern and southern Europe. The dangers of extreme heat, particularly in urban areas, are well known^1,2^. Unusually, many governments in western Europe took the step of releasing near-real-time estimates of the numbers of excess deaths associated with the extreme heat, underscoring both the severity of the temperatures and the high level of associated risk (for example, https://www.santepubliquefrance.fr/en/press/5764-excess-deaths-france-during-heat-wave-june-17-july-2 and https://www.gov.uk/government/publications/interim-heat-mortality-monitoring-report-england-may-and-june-2026/).

Europe is known to be the fastest-warming continent^3^, being an estimated 2.5 °C warmer in 2026 than it would have been in a pre-industrial climate. By contrast, the world as a whole is almost 1.5 °C warmer, with around 1.4 °C of that warming attributable to human activity, primarily the burning of fossil fuels^4^. Several studies have shown that human-caused warming has contributed significantly to heat-related mortality, with most heat-related excess deaths in recent years found to be attributable to anthropogenic warming^5–7^. In this study, we extend the analysis of ref. 7 to assess the impact of human-induced climate change on heat-related mortality in 854 European cities during the record-breaking summer of 2026. Using established methods for probabilistic attribution of heat and modelling of heat-related excess deaths^1,7,8^, we quantify the expected number of heat-related excess deaths across 854 cities in Europe during the summer months and the number and proportion of these deaths attributable to anthropogenic climate change.

Figure 1a shows the daily mean temperatures averaged over the 854 cities in the study region duringthe summer months (June–August) of 2026, under both the factual (observed) and counterfactual (without human-caused warming) scenarios. Over all 854 cities, the summer was on average around 2.4 °C hotter than the 1991–2020 climatology, although on individual days the anomaly (the departure from typical mean temperatures for the time of year) reached 7.3 °C (Fig. 1a and Extended Data Table 1). The season as a whole was most unusually warm over western and southern Europe (respectively, anomalies of 2.9 °C and 2.6 °C), although the highest daily anomalies occurred in western and eastern Europe, with some countries enduring days that exceeded the 1991–2020 climatology by as much as 12 °C.

We estimate 50,391 heat-related excess deaths (95% empirical confidence interval (eCI) 46,026–53,878) during the summer months of June to August over the 854 cities, which represent a combined population of almost 173 million residents in 2026 (Table 1). The highest mortality is estimated among individuals aged 85 years or older (22,502 heat-related excess deaths, 95% eCI 20,620–24,414; Extended Data Table 2), but even among the 20–44 years age group we find around 833 heat-related excess deaths (95% eCI 628–1053). Although this analysis focuses on the summer months of June–August, we also extended the analysis to May, finding a further 1,508 heat-related deaths between 21 May and 31 May (95% eCI 1,305–1,725; Extended Data Table 3).

We observe marked spatial variabilities in modelled heat-related mortality across the continent. In northern Europe, only the most extreme heat events are associated with high mortality rates (Extended Data Figs. 1 and 2) and rates are low compared with other regions (129 per million population after standardizing for age, 95% eCI 105–153; Table 1), although substantially higher in 2026 than historical estimates^1^. In western and eastern Europe, high mortality is also mainly associated with individual heat events, but temperatures are generally hotter, leading to higher rates (277, 95% eCI 241–308; 250, 95% eCI 181–307). In southern Europe, dangerously hot temperatures persisted from mid-June onwards, leading to the highest excess heat-mortality rate (380, 95% eCI 334–430). However, individual countries experienced much higher rates, with the highest modelled mean age-standardized excess mortality per million population occurring in Malta (716; 95% eCI 535–874), Italy (561; 95% eCI 499–634), Hungary (537; 95% eCI 391–681) and Croatia (461; 95% eCI 370–543); Fig. 1c and Table 1.

Although Europe as a whole is today around 2.5 °C warmer than it would be in the absence of human-caused warming, spatial patterns of attributable warming vary across the continent, with the strongest trends observed in eastern and southern Europe (Eastern Europe 2.8 °C, 95% eCI 2.0–3.5 °C; Southern Europe 2.6 °C, 95% eCI 1.9–3.2 °C; Extended Data Table 1). Over all 854 cities, we estimate 32,619 heat-related deaths (95% eCI 26,078–38,237) attributable to anthropogenic climate change over the study period, corresponding to 65% of the total mortality burden (95% eCI 53–73%; Table 1). Regionally, southern Europe experienced the highest proportion of deaths attributable to human-caused warming (69%; 95% eCI 53–80%) and northern Europe the lowest (59%; 95% eCI 43–71%). In all countries other than Malta, where uncertainty about the rate of warming is extremely high, more than 50% of the modelled deaths were found to be attributable to anthropogenic warming (Fig. 1d and Table 1), with the highest proportions in cities across Estonia (81%; 95% eCI 54–98%), Bulgaria (81%; 95% eCI 60–93%) and Finland (80%; 95% eCI 50–96%).

Almost one-third of the modelled heat-related excess deaths occurred between 17 June and 1 July, when daily mean temperatures over the 854 cities were on average 5.3 °C above the 1991–2020 mean (Extended Data Fig. 1 and Extended Data Table 3). During this period a slightly lower proportion of the deaths were attributable to anthropogenic warming (57%; 95% eCI 42–68%). This reflects the severity of the heatwave: in general, the proportion of modelled deaths attributable to human-caused warming is lower during more extreme heat events, because the high temperatures reached would have been dangerously hot even in a pre-industrial climate without human-caused warming. This is illustrated by the fact that the highest proportion of attributable deaths occurred in Estonia, Bulgaria and Finland; three countries that had comparably cool summers, but would have barely reached the mortality threshold in the counterfactual climate, meaning that the small number of modelled deaths are almost entirely due to human-caused warming.

Although the absolute numbers of modelled heat-related excess deaths are, to some extent, sensitive to assumptions about population size and age composition (Methods, ‘Sensitivity analysis’), when tested using mortality rates based on historic population and demographic estimates the proportions attributable to anthropogenic warming are comparable. However, we note that the proportion of heat-related deaths attributable to human activity is likely to be sensitive to the attribution model used, in this case a shift in monthly mean temperatures, which is expected to be a conservative estimate of the true warming (Supplementary Information, section 2 of ref. 7).

Our estimate of 50,391 heat-related excess deaths in summer 2026 indicates a substantially larger mortality burden than estimates across the same set of cities using the same analytical framework for other benchmark summers of 2022 (29,480, 95% eCI 26,643–32,006) and 2003 (28,462, 95% eCI 25,709–31,199) and more than double the estimate reported for 2025 (24,572, 95% eCI 22,064–26,867) (Extended Data Table 4, reproduced from ref. 7). Moreover, the number of additional deaths attributable to human-caused warming in 2026 is of comparable magnitude to the total modelled mortality during the summers of 2022 and 2003.

This analysis covers urban populations only, representing almost 40% of the population of the 30 countries. Consequently, the total heat-related mortality burden across Europe will be larger, although the magnitude cannot be inferred directly from our results. A rapid report estimated 20,390 excess heat-related deaths during 22–28 June 2026 across the whole of Europe^9^, whereas our modelling finds 9,585 over the same period (95% eCI 8,815–10,310; Extended Data Table 5). Although that study includes a slightly different set of countries to those used in this study, a simple scaling of that result by 40% would result in 8,156 deaths, only slightly lower than our estimate, indicating that the two results are broadly compatible despite their different assumptions and approaches.

Several government agencies took the unusual step of issuing near-real-time estimates of the mortality associated with the most intense heat events, underscoring the severity of the threat to public health. As in our analysis, the highest mortality was reported during the extreme heatwave over western Europe in late June: Santé Publique of France reported 5,764 heat-related excess deaths between 17 June and 2 July, whereas the UK Health Security Agency estimated that 2,124 heat-related excess deaths occurred across England and Wales from 21 June to 28 June (Extended Data Table 5). The Robert Koch Institute of Germany estimates 16,000 heat-related excess deaths from calendar week 15 (6 April) to week 36 (6 September), whereas the Mortality Monitoring System of Spain reports 5,123 heat-related excess deaths between June and August. Direct comparison of our results with these numbers is difficult not only because of the different spatial coverage but also because of the very different methods used, with official estimates often derived by comparing registered deaths with the expected number estimated from a baseline period that may include some human-caused warming. Nonetheless, the official numbers are broadly consistent with our modelled estimates after accounting for the partial coverage of our study region.

Our estimates of heat-related mortality burden are also broadly consistent with previous studies in terms of spatial pattern. In line with previous studies the largest mortality burden was found in southern Europe: in ref. 10 a southwest–northeast corridor of highest mortality from southern Portugalthrough France to Germany duringthe summer of 2003 was identified, and in refs. 1,11 the largest burdens and rates in 2022 were reported in southern European countries. This probably reflects a combination of sustained extreme heat exposure and greater population vulnerability to heat, due to factors such as prevalence of chronic diseases, demographics, socioeconomic deprivation, air-pollution exposure and green space coverage^1,2,11^.

Although estimates of the absolute heat-related mortality burden differ slightly across studies owing to differences in population coverage, time period and model assumptions, the proportion of deaths attributable to anthropogenic climate change appears more consistent across analyses. Our estimate of 65% (95% eCI 53–73%) is slightly higher than estimates for the summer of 2022 in Europe (56%) and Switzerland (60%)^12,13^, although these estimates still fall well within our confidence intervals. This slightly higher attributable proportion may reflect not only recent warming trends^7^, but also greater urban heat exposure in our study region, related to the urban heat-island effect and higher population density.

The modelling framework applied here assumes that vulnerability to heat is constant throughout the year and depends only on daily mean temperatures. However, in many countries the most extreme event occurred in June, relatively early in the summer when people are less likely to be acclimatized to extreme heat, and was characterized by both extremely high humidity and unusually high night-time temperatures^14^. All three of these factors may contribute to increased mortality and may therefore result in underestimation of the true mortality burden during the event.

The exposure–response functions (ERFs) used to estimate heat-related excess deaths were derived using data from 2000 to 2019 and therefore do not capture potential post-2019 changes in heat vulnerability, including changes in demographic composition, population frailty, healthcare, adaptation or other postpandemic changes in population health. Estimating time-varying exposure–response relationships to account for adaptation or changing heat vulnerability is beyond the scope of this study ; however, these static ERFs allow us to compare our results directly with those of ref. 7 and so focus on the contribution of both the extreme temperatures experienced in 2026 and the effect of anthropogenic warming.

Our analysis indicates that 2026 is likely to have been the most dangerously hot summer that Europe has experienced to date, with modelled heat-related excess deaths across these 854 cities substantially higher than in either 2003 or 2022. Partly this is because 2026 was an unusually hot summer even in the current climate, with many European countries experiencing several heatwaves throughout the season. In comparison, the heat-related mortality burden of 2003 was mostly concentrated around the exceptional heatwave of early August. However, human-caused warming driven primarily by the burning of fossil fuels accounts for around two-thirds of the modelled deaths over the summer, adding a mortality burden that may be outpacing adaptation^15,16^. Under currently implemented policies, a further 1.4 °C of warming is projected by the end of the century; without rapid progress towards net zero emissions to slow the rate of warming, the health burden arising from human-caused warming will continue to increase, placing growing pressure on public health systems.

## Methods

This study uses a two-stage methodological framework to model heat-related excess deaths and disentangle the contribution of anthropogenic warming. The first step uses a probabilistic attribution approach to estimate local warming due to anthropogenic climate change and by subtracting the estimated warming from observed (factual) temperatures, to simulate counterfactual temperatures representative of a world without human-caused warming. The second stage integrates these temperature estimates with published exposure–response relationships for 854 cities in Europe, using recent mortality rates and population estimates to estimate heat-related excess deaths under both the factual and counterfactual scenarios. For a full description of the data and methods used and a discussion of the strengths and limitations of the approach, see ref. 7.

### Probabilistic climate attribution and counterfactual temperatures

Factual temperatures are taken from ERA5-Land reanalysis^17^, with counterfactuals obtained by subtracting the attributable local warming from the factual temperatures.

The degree of local warming attributable to human activity is estimated using an established probabilistic attribution methodology^8,18^. First, time series of daily mean temperatures in each of the 854 cities during the Northern Hemisphere summer months are retrieved from gridded temperature data products: both from observations-based datasets (ERA5-Land reanalysis from 1950 to 2026 and E-Obs interpolated observations from 1920 to 2026, v33.0 + pre1950^19^) and from 36 distinct models from the Coupled Model Intercomparison Project Phase 6 (CMIP6) multimodel ensemble, using historical forcings from 1850 to 2014 and Shared Socioeconomic Pathway (SSP) 5-8.5 forcings from 2015 to 2030^20^. For all datasets we use a regression model to estimate the local warming in each city in response to each degree of global warming and hence to estimate the amount of local warming associated with the 1.4 °C of human-caused global warming estimated to have occurred by 2026^4^. These estimates are combined into an observational synthesis and a climate model synthesis using the algorithm described in ref. 18 and we take the unweighted mean of these to avoid over-weighting towards climate models, which are known to underestimate local warming in Europe^21^.

### Health impact assessment

Health impacts were estimated using cumulative ERFs derived from distributed lag nonlinear models, trained on historical mortality data from 2000 to 2019 in 232 EU cities; attributable numbers are generated using an established procedure used in previous health impact assessments^1,22^. The resulting ERFs are age specific (years 20–44, 45–64, 65–74, 75–84, 85+), accounting for the unequal burden in older populations. These functions are combined with the factual and counterfactual temperature time series to estimate relative risk of heat mortality under the two scenarios. We used historical mortality data from Eurostat and population data from WorldPop to estimate mortality rates for 2026 across the 854 cities and integrate them with the resulting relative risks to estimate the number and proportion of heat-related excess deaths attributable to human-induced climate change. For full details of the methods and data used, see Supplementary Information, section 1 of ref. 7.

### Uncertainty propagation

The final estimates of the heat-mortality burden combine the uncertainty from both the counterfactual temperatures and the ERFs. We construct 1,000 spatially coherent samples of counterfactual temperatures by sampling from a multivariate normal distribution with the same marginal means and variances as the synthesized attributable warming in each city and calendar month, and the same correlation matrix over all cities and calendar months as the attributable warming in the factual dataset. Uncertainty in the ERFs is assessed through Monte Carlo simulation by generating 1,000 samples from the parameters of the fitted meta-regression model that was then used to predict ERFs^23^. This sampling procedure preserves the dependence between cities and age groups arising from shared meta-regression parameters. For each ERF draw, we calculated the heat-related excess deaths for the factual and one randomly selected counterfactual temperature series—this is a computationally efficient equivalent to calculating all possible combinations of counterfactual temperatures and ERFs and then randomly sampling 1,000 instances. The difference and the proportion of deaths due to anthropogenic climate change were then calculated from the within-draw difference between factual and counterfactual mortality. Medians and 95% empirical confidence intervals for all quantities are computed from these 1,000 samples and thus capture uncertainty about both the rate of local warming and the ERFs.

### Sensitivity analysis

To test the robustness of the results to the choice of population and mortality rates used in the analysis presented here, we repeated the analysis using the same estimated populations and mortality rates as in ref. 7, which followed ref. 1 in using the mean population and annual mortality rates from 2000 to 2019. Although the absolute estimates of excess deaths are generally slightly lower because of the historically smaller population sizes, the proportion of heat-related deaths attributable to anthropogenic warming remains stable (Extended Data Table 6).

This sensitivity analysis also allows us to directly compare our estimates of heat-related mortality during summer 2026 with estimates for previous years from ref. 7, which found that the summers with the highest heat-related mortality were 2022 (29,480 deaths under the factual scenario; 95% eCI 26,643–32,006) and 2003 (28,462 deaths under the factual scenario; 95% eCI 25,709–31,199); Extended Data Table 4. Under the same assumptions about population size and annual mortality rates, we find an estimated 45,492 deaths during summer 2026 (95% eCI 41,429–48,719), around 50% higher than in either 2022 or 2003. The modelled number of heat-related deaths attributable to human-caused warming in 2026 (29,330; 95% eCI 23,495–34,420) is of similar magnitude to the total number of deaths due to extreme heat in either 2022 or 2003 (Extended Data Table 4).

### Reporting summary

Further information on research design is available in the Nature Portfolio Reporting Summary linked to this article.

## Extended Data

**Extended Data Fig. 1:**
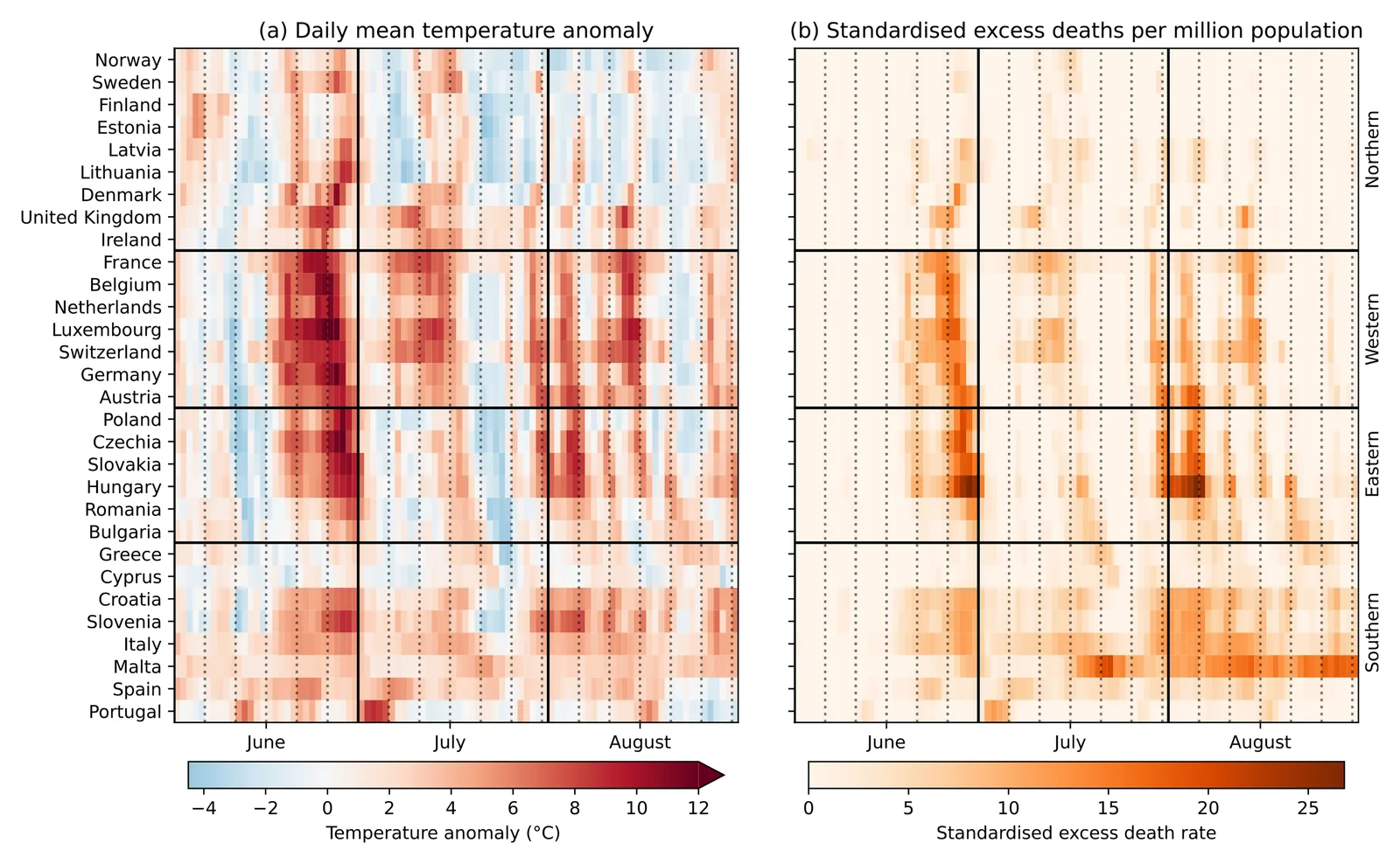
Daily temperature exposure and mortality burden across 30 European countries. Heatmaps of (**a**) daily mean temperature anomaly with respect to 1991-2020 climatology and (**b**) standardised excess deaths per million population between June and August 2026, averaged over all cities in each country.

**Extended Data Fig. 2:**
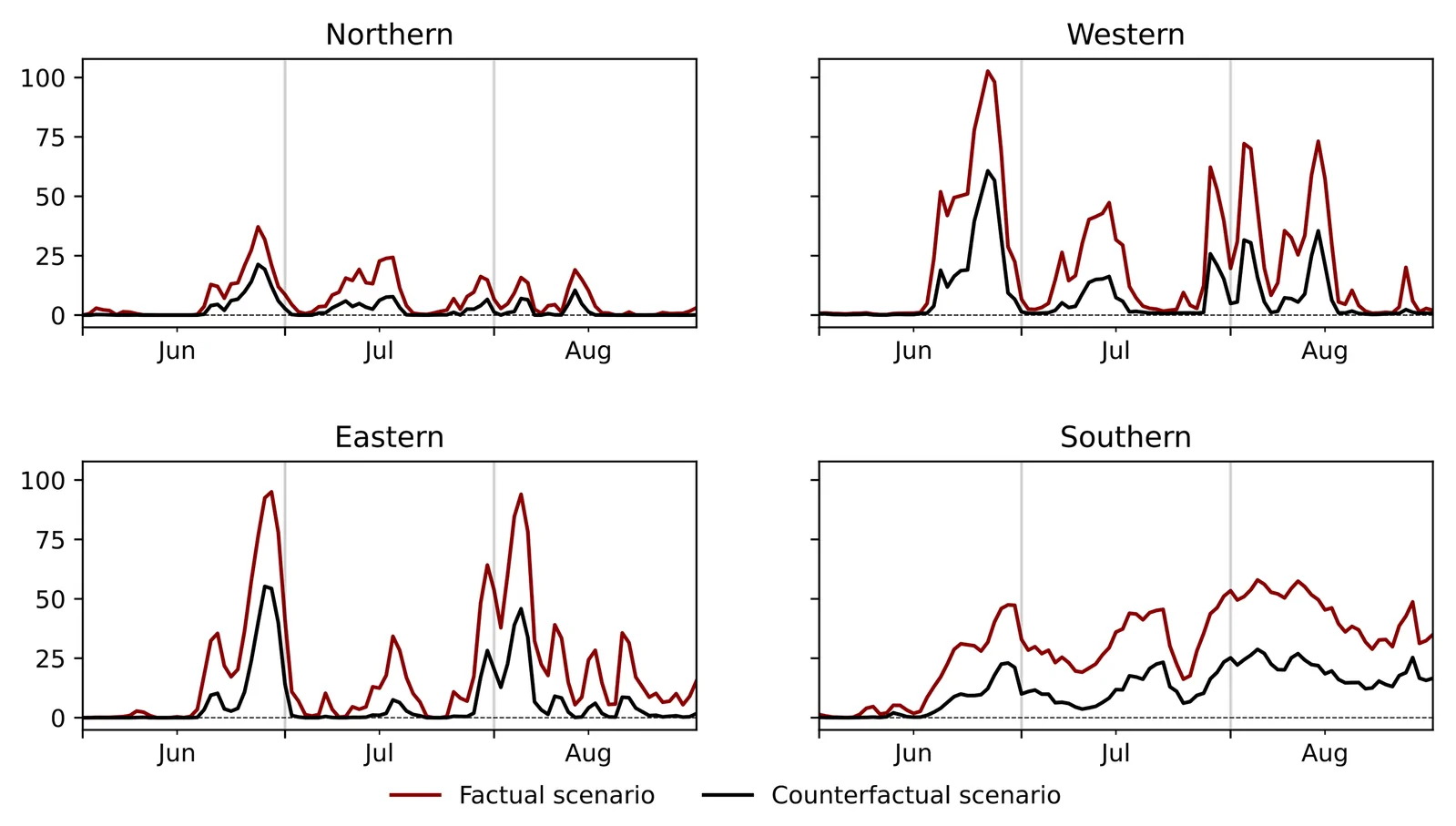
Varying patterns of regional heat mortality throughout the summer. Standardised daily heat-related excess deaths per million population under the factual and counterfactual scenarios, aggregated over all cities within each region. See Extended Data Table 1 for details of regions.

**Extended Data Table 1:** Summer mean/maximum daily mean temperature and temperature anomaly, and attributable warming averaged over all cities in each country

Region	Country	N. cities	Mean / max JJA temp	Mean / max JJA anomaly	Attributable warming
Northern	Norway	4	13.8 / 19.0	0.2 / 5.2	1.7 (1.1 - 2.4)
Northern	Sweden	14	17.3 / 23.6	1.0 / 7.7	1.9 (1.1 - 2.6)
Northern	Finland	9	16.4 / 22.1	0.4 / 5.5	2.0 (1.2 - 2.8)
Northern	Estonia	3	17.2 / 22.3	0.4 / 5.6	2.0 (1.2 - 2.9)
Northern	Latvia	10	17.6 / 23.9	0.3 / 7.1	2.1 (1.4 - 3.0)
Northern	Lithuania	6	17.9 / 26.0	0.3 / 8.9	2.2 (1.5 - 3.0)
Northern	Denmark	4	17.6 / 26.6	1.1 / 10.8	1.7 (1.1 - 2.3)
Northern	United Kingdom	135	18.0 / 24.9	2.5 / 9.0	1.8 (1.1 - 2.5)
Northern	Ireland	5	16.6 / 22.5	1.8 / 7.7	1.2 (0.5 - 2.0)
	**Northern Europe**	**190**	**17.7 / 22.9**	**2.0 / 7.4**	**1.8 (1.2 - 2.4)**
Western	France	72	23.0 / 29.3	3.8 / 10.4	2.7 (1.9 - 3.5)
Western	Belgium	15	20.3 / 28.8	2.8 / 11.4	2.4 (1.6 - 3.3)
Western	Netherlands	47	19.7 / 28.1	2.3 / 11.2	2.2 (1.6 - 2.8)
Western	Luxembourg	1	20.8 / 29.7	3.4 / 12.2	2.7 (1.9 - 3.5)
Western	Switzerland	12	21.4 / 27.1	4.0 / 9.5	2.9 (2.2 - 3.6)
Western	Germany	127	20.6 / 29.8	2.5 / 12.0	2.5 (1.8 - 3.2)
Western	Austria	6	20.9 / 27.5	3.1 / 9.6	3.1 (2.2 - 4.0)
	**Western Europe**	**280**	**21.1 / 28.3**	**2.9 / 10.2**	**2.5 (1.9 - 3.2)**
Eastern	Poland	68	19.5 / 29.4	1.1 / 11.5	2.6 (1.9 - 3.2)
Eastern	Czechia	18	20.4 / 29.8	2.2 / 12.2	2.8 (2.1 - 3.6)
Eastern	Slovakia	8	21.5 / 29.7	2.4 / 10.7	3.0 (2.1 - 3.9)
Eastern	Hungary	19	24.3 / 31.4	2.9 / 9.9	2.9 (1.8 - 3.9)
Eastern	Romania	35	22.4 / 28.4	1.3 / 7.8	2.9 (2.0–4.0)
Eastern	Bulgaria	18	23.0 / 27.1	1.3 / 5.8	2.8 (1.9 - 3.8)
	**Eastern Europe**	**166**	**21.2 / 28.4**	**1.6 / 9.3**	**2.8 (2.0 - 3.5)**
Southern	Greece	14	25.2 / 28.8	0.9 / 3.7	2.1 (1.4 - 3.0)
Southern	Cyprus	3	28.5 / 32.3	0.3 / 2.7	2.1 (0.2 - 3.8)
Southern	Croatia	7	24.9 / 29.4	2.6 / 6.9	3.1 (2.1 - 3.9)
Southern	Slovenia	2	22.6 / 28.9	3.0 / 8.8	3.2 (2.0 - 4.3)
Southern	Italy	87	26.3 / 29.3	3.2 / 5.8	2.7 (1.8 - 3.6)
Southern	Malta	1	27.3 / 30.7	2.6 / 5.1	0.9 (-1.8 - 3.5)
Southern	Spain	90	25.2 / 28.3	2.6 / 6.1	2.6 (1.9 - 3.3)
Southern	Portugal	14	21.8 / 28.5	1.2 / 8.8	1.8 (1.1 - 2.5)
	**Southern Europe**	**218**	**25.4 / 28.1**	**2.6 / 5.1**	**2.6 (1.9 - 3.2)**
	**Europe**	**854**	**21.5 / 26.1**	**2.4 / 7.3**	**2.5 (1.9 - 2.9)**

List of countries in each region with number of cities used in the study; mean and maximum daily summer temperature and anomaly with respect to 1991-2020, averaged over all cities (ERA5-Land); mean attributable warming of June-August temperatures averaged over all cities in each country/region, with 95% empirical confidence interval given in parentheses. All temperatures in °C.

**Extended Data Table 2:** Modelled mortality per age group

Age group	Population	Heat deaths	N. attributable	Prop. Attributable
20-44	71,616,048	833 (628, 1,053)	419 (283, 559)	0.50 (0.39, 0.62)
45-64	56,935,376	5,189 (4,452, 5,832)	3,126 (2,487, 3,755)	0.61 (0.49, 0.70)
65-74	22,676,014	7,646 (6,861, 8,346)	4,848 (3,924, 5,667)	0.64 (0.51, 0.72)
75-84	15,482,080	14,182 (12,979, 15,203)	9,181 (7,333, 10,732)	0.65 (0.53, 0.73)
85+	6,008,162	22,502 (20,620, 24,414)	14,984 (11,829, 17,634)	0.67 (0.54, 0.76)
**Total**	**172,717,680**	**50,391 (46,026, 53,878)**	**32,619 (26,078, 38,237)**	**0.65 (0.53, 0.73)**

Population per age group, median and 95% empirical confidence intervals of modelled number of heat-related excess deaths in the factual scenario; absolute number of deaths attributable to human-caused warming; and proportion of deaths attributable to human-caused warming during June-August 2026 in 854 European cities, aggregated per age group.

**Extended Data Table 3:** Mean/maximum daily mean temperature and temperature anomaly during heat events, along with modelled mortality and proportion attributable to anthropogenic warming

Period	Mean / max daily temperature	Mean / max daily anomaly	Excess deaths	N. attributable	Prop. attributable
May 5 – May 31	19.5 / 21.6	4.1 / 6.1	1,508 (1,305, 1,725)	1,017 (644, 1,282)	0.67 (0.43, 0.82)
June 17 – July 1	23.7 / 26.1	5.3 / 7.3	14,539 (13,349, 15,592)	8,262 (6,145, 10,198)	0.57 (0.42, 0.68)
July 5 – July 18	22.6 / 23.5	3.1 / 3.8	9,314 (8,582, 10,091)	6,437 (4,723, 7,673)	0.69 (0.51, 0.81)
July 28 – August 6	23.7 / 25.2	3.0 / 4.5	9,400 (8,481, 10,162)	6,001 (4,559, 7,191)	0.64 (0.49, 0.75)
August 8 – August 18	23.0 / 24.6	3.0 / 4.9	9,082 (8,336, 9,768)	6,108 (4,612, 7,341)	0.67 (0.51, 0.79)

Daily mean/max temperature and temperature anomaly (relative to 1991-2020 climatology for each day of the year) over all 854 cities during selected heat periods, along with modelled number of heat-related excess deaths, absolute number of deaths attributable to human-caused warming, and proportion of deaths due to human-caused warming.

**Extended Data Table 4:** Annual mortality estimates across the 854 cities for the five years with the highest estimated heat mortality between 2000 and 2025

Year	Factual	Counterfactual	N. attributable	Prop. attributable
2022	29,480 (26,643, 32,006)	12,116 (9,121, 15,963)	17,215 (13,570, 20,173)	0.59 (0.46, 0.68)
2003	28,462 (25,709, 31,199)	16,588 (13,673, 19,832)	11,867 (9,172, 14,180)	0.42 (0.33, 0.50)
2024	26,005 (22,858, 28,737)	8,860 (6,003, 12,810)	17,111 (13,188, 20,055)	0.66 (0.52, 0.76)
2025	24,572 (22,064, 26,867)	7,760 (5,385, 11,004)	16,768 (13,620, 19,321)	0.68 (0.56, 0.77)
2015	23,673 (20,946, 25,841)	11,101 (8,420, 14,457)	12,537 (9,361, 15,049)	0.53 (0.40, 0.63)

Median and 95% empirical confidence intervals of total number of expected heat-deaths under the factual and counterfactual scenarios, and the absolute number and proportion due to human-made climate change. Reproduced from ref. 7, Springer Nature Ltd.

**Extended Data Table 5:** Comparison of our mortality estimates with other reported numbers

Region	Period	Reported	Our estimate	Coverage
Europe^1^	June 22 – June 28	20,390	9,585 (8,815, 10,310)	40%
France^2^	June 17 – July 2	5,764	2,305 (1,990, 2,606)	30%
UK^3^	June 21 – June 28	2,124	1,375 (1,122, 1,611)	50%
Germany^4^	May 1 – August 31*	16,000	6,477 (5,478, 7,314)	30%
Spain^5^	June 1 – August 31	5,123	6,824 (5,744, 7,984)	40%

Reported regional estimates of heat-related mortality for specific periods, and our estimates over the same region and period. ‘Coverage’ denotes the approximate proportion of each region’s population represented within the study region. *RKI report covers calendar weeks 15 (April 6th) to 36 (September 6th). Sources: 1^9^. 2. https://www.santepubliquefrance.fr/en/press/5764-excess-deaths-france-during-heat-wave-june-17-july-2; 3. https://www.gov.uk/government/publications/interim-heat-mortality-monitoring-report-england-may-and-june-2026/; 4. Robert Koch Institute https://edoc.rki.de/handle/176904/13926#; 5. https://momo.isciii.es/panel_momo/.

**Extended Data Table 6:** As Table 1, but using historic mean population and mortality rates from ^7^ to test the sensitivity of the results to these assumptions

Country/region	Population	Excess deaths	Sth. Excess	N. attributable	Prop. Attributable
Norway	829,551	19 (6, 31)	29 (9, 48)	11 (4, 21)	0.63 (0.34, 0.82)
Sweden	2,807,484	122 (77, 165)	45 (28, 61)	83 (49, 123)	0.70 (0.48, 0.83)
Finland	1,460,602	25 (12, 46)	18 (9, 34)	19 (8, 34)	0.80 (0.50, 0.96)
Estonia	440,155	13 (4, 33)	32 (10, 79)	11 (4, 23)	0.80 (0.52, 0.98)
Latvia	874,434	89 (24, 175)	101 (26, 198)	58 (14, 120)	0.67 (0.42, 0.83)
Lithuania	1,033,748	115 (22, 210)	114 (23, 207)	60 (8, 119)	0.55 (0.30, 0.73)
Denmark	917,663	59 (36, 83)	72 (45, 101)	34 (19, 51)	0.58 (0.39, 0.72)
United Kingdom	26,545,907	3,616 (2,890, 4,329)	154 (123, 185)	2,098 (1,370, 2,755)	0.59 (0.41, 0.72)
Ireland	1,090,316	51 (23, 78)	69 (33, 105)	30 (9, 52)	0.61 (0.26, 0.79)
**Northern Europe**	**35,999,861**	**4,084 (3,313, 4,889)**	**128 (104, 153)**	**2,410 (1,642, 3,111)**	**0.59 (0.43, 0.71)**
France	17,962,199	6,282 (5,392, 7,141)	335 (287, 381)	4,012 (3,037, 4,978)	0.65 (0.51, 0.75)
Belgium	2,455,390	646 (522, 753)	267 (215, 310)	365 (253, 476)	0.57 (0.41, 0.69)
Netherlands	5,644,325	961 (706, 1,213)	187 (138, 235)	517 (360, 677)	0.54 (0.39, 0.67)
Luxembourg	70,074	21 (16, 25)	372 (292, 445)	12 (9, 16)	0.60 (0.46, 0.71)
Switzerland	1,977,029	862 (666, 1,023)	447 (345, 530)	586 (427, 744)	0.69 (0.54, 0.80)
Germany	23,406,685	6,450 (5,461, 7,277)	265 (225, 299)	3,741 (2,770, 4,585)	0.58 (0.45, 0.68)
Austria	1,949,160	640 (526, 759)	365 (300, 432)	417 (309, 542)	0.66 (0.50, 0.78)
**Western Europe**	**53,464,863**	**15,861 (13,748, 17,579)**	**292 (253, 324)**	**9,660 (7,347, 11,705)**	**0.61 (0.48, 0.71)**
Poland	10,396,899	1,986 (1,434, 2,446)	215 (155, 263)	1,128 (774, 1,497)	0.58 (0.45, 0.69)
Czechia	2,522,881	612 (406, 783)	282 (190, 358)	368 (236, 496)	0.61 (0.49, 0.71)
Slovakia	877,322	239 (157, 308)	370 (245, 474)	149 (87, 204)	0.63 (0.47, 0.75)
Hungary	2,806,295	1,035 (735, 1,310)	401 (285, 505)	628 (396, 858)	0.61 (0.43, 0.75)
Romania	6,010,799	1,348 (895, 1,802)	258 (171, 344)	996 (670, 1,360)	0.75 (0.59, 0.88)
Bulgaria	2,596,877	586 (390, 818)	251 (168, 348)	461 (303, 643)	0.80 (0.59, 0.93)
**Eastern Europe**	**25,211,073**	**5,843 (4,143, 7,250)**	**263 (188, 324)**	**3,731 (2,597, 4,883)**	**0.65 (0.52, 0.76)**
Greece	3,401,064	578 (417, 725)	160 (116, 201)	417 (296, 537)	0.74 (0.55, 0.87)
Cyprus	457,113	53 (34, 69)	156 (101, 205)	39 (2, 61)	0.78 (0.04, 0.99)
Croatia	1,101,531	597 (480, 708)	580 (465, 692)	378 (258, 504)	0.64 (0.45, 0.79)
Slovenia	308,320	127 (99, 157)	439 (339, 542)	78 (52, 107)	0.62 (0.44, 0.77)
Italy	17,186,805	11,565 (10,269, 13,006)	575 (510, 647)	7,612 (5,421, 9,542)	0.66 (0.48, 0.79)
Malta	170,361	102 (76, 125)	739 (566, 904)	30 (-80, 97)	0.31 (-0.79, 0.89)
Spain	17,714,878	6,221 (5,258, 7,280)	330 (279, 385)	4,691 (3,523, 5,785)	0.76 (0.60, 0.86)
Portugal	3,457,781	454 (364, 579)	127 (102, 162)	232 (150, 324)	0.51 (0.35, 0.66)
**Southern Europe**	**43,797,852**	**19,717 (17,361, 22,297)**	**411 (362, 466)**	**13,468 (10,098, 16,541)**	**0.68 (0.52, 0.80)**
**Europe**	**158,473,649**	**45,492 (41,429, 48,719)**	**291 (265, 312)**	**29,330 (23,495, 34,420)**	**0.65 (0.52, 0.73)**

Population per country, median and 95% empirical confidence intervals of modelled number of heat-related excess deaths in the factual scenario; standardised excess deaths per million population; absolute number of deaths and proportion of deaths attributable to human-made climate change during June-August 2026 in 854 European cities, using historic mortality rates and population estimates, aggregated over thirty countries.

## Supplementary Material

**Supplementary information** The online version contains supplementary material available at https://doi.org/10.1038/s44360-026-00220-z.

Supplementary Files

## Figures and Tables

**Fig. 1:**
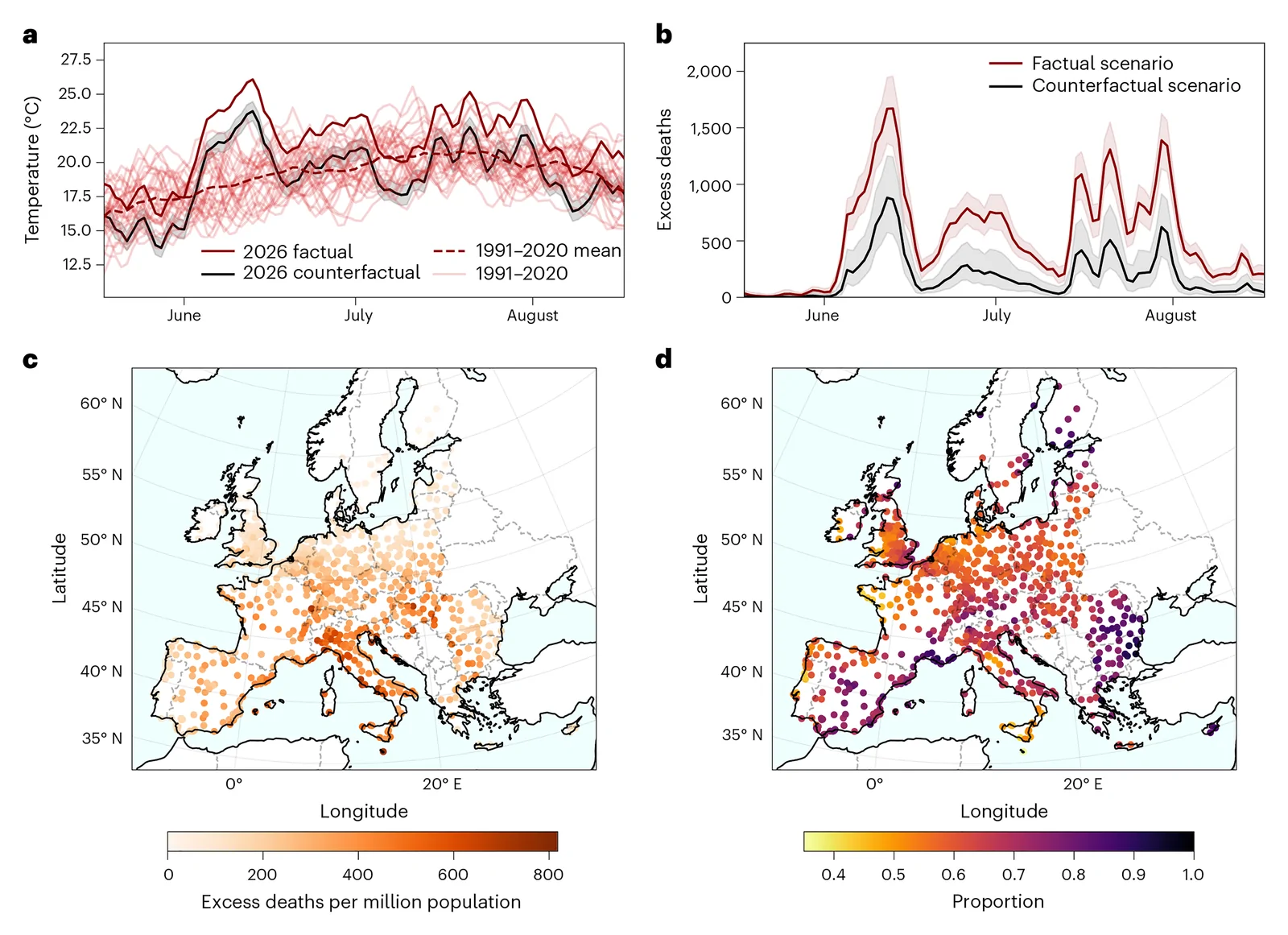
Daily mean temperatures, modelled heat-related excess deaths and proportion of heat-related excess deaths attributable to human-caused warming across Europe during summer 2026. **a**, Factual and median counterfactual daily mean temperatures averaged over all 854 cities during the summer months (June–August) of 2026, with 1991–2020 daily mean temperatures and climatology for comparison and 95% confidence interval for counterfactual temperatures indicated by shaded area. **b**, Daily estimated heat-related excess deaths under the factual and counterfactual scenarios, with 95% confidence intervals indicated by shaded areas. **c**, Median age-standardized death rate per million inhabitants in each of the 854 cities between June and August 2026. **d**, Median proportion of estimated heat-related excess deaths between June and August 2026 attributable to anthropogenic warming in each of the 854 cities. Basemap data in **c** and **d** from Natural Earth (https://www.naturalearthdata.com/).

**Table 1:** Heat-related excess deaths during June–August 2026 in 854 European cities of 30 countries

Country/region	Population^a^	Excess deaths^b^	Standardized excess^c^	Number attributable^d^	Proportion attributable^e^
Norway	1,082,996	20 (6, 33)	28 (9, 44)	12 (4, 22)	0.62 (0.34, 0.82)
Sweden	3,536,039	127 (81, 171)	39 (25, 53)	87 (52, 129)	0.70 (0.49, 0.83)
Finland	1,806,484	29 (14, 54)	16 (8, 31)	22 (9, 40)	0.80 (0.50, 0.96)
Estonia	464,034	13 (5, 33)	26 (9, 65)	11 (4, 23)	0.81 (0.54, 0.98)
Latvia	750,258	96 (25, 192)	132 (32, 265)	64 (15, 137)	0.69 (0.44, 0.85)
Lithuania	973,935	102 (21, 183)	115 (23, 204)	54 (8, 105)	0.56 (0.32, 0.73)
Denmark	1,684,793	99 (63, 138)	72 (46, 100)	58 (32, 85)	0.58 (0.40, 0.73)
UK	29,959,591	4,354 (3,491, 5,227)	158 (127, 190)	2,535 (1,668, 3,310)	0.59 (0.41, 0.71)
Ireland	1,506,389	60 (26, 92)	50 (22, 77)	35 (10, 61)	0.60 (0.24, 0.78)
**Northern Europe**	**41,764,519**	**4,891 (3,965, 5,833)**	**129 (105, 153)**	**2,881 (1,971, 3,739)**	**0.59 (0.43, 0.71)**
France	19,699,428	7,268 (6,228, 8,243)	336 (288, 382)	4,661 (3,530, 5,774)	0.65 (0.52, 0.75)
Belgium	2,827,900	693 (562, 803)	243 (198, 281)	393 (272, 513)	0.58 (0.41, 0.69)
Netherlands	7,852,432	1,329 (981, 1,683)	184 (136, 232)	721 (502, 938)	0.55 (0.39, 0.67)
Luxembourg	109,239	29 (22, 34)	318 (248, 378)	17 (12, 22)	0.60 (0.46, 0.71)
Switzerland	2,151,882	934 (728, 1,106)	386 (299, 457)	636 (464, 804)	0.69 (0.54, 0.80)
Germany	25,014,583	6,340 (5,367, 7,136)	240 (203, 271)	3,685 (2,731, 4,501)	0.59 (0.45, 0.68)
Austria	2,378,237	783 (642, 928)	339 (277, 401)	515 (380, 662)	0.67 (0.51, 0.79)
**Western Europe**	**60,033,701**	**17,349 (15,132, 19,254)**	**277 (241, 308)**	**10,643 (8,123, 12,872)**	**0.62 (0.49, 0.71)**
Poland	10,372,157	2,174 (1,569, 2,673)	199 (144, 245)	1,245 (855, 1,639)	0.58 (0.45, 0.69)
Czechia	2,592,557	701 (472, 896)	267 (180, 340)	425 (276, 569)	0.62 (0.50, 0.71)
Slovakia	925,601	297 (195, 379)	335 (224, 429)	187 (111, 254)	0.64 (0.48, 0.76)
Hungary	2,742,706	1,004 (703, 1,275)	537 (391, 681)	615 (387, 843)	0.62 (0.43, 0.75)
Romania	4,760,539	1,105 (730, 1,472)	248 (165, 328)	819 (552, 1,118)	0.75 (0.60, 0.88)
Bulgaria	2,748,521	618 (411, 868)	236 (156, 331)	489 (320, 689)	0.81 (0.60, 0.93)
**Eastern Europe**	**24,142,081**	**5,935 (4,256, 7,331)**	**250 (181,307)**	**3,782 (2,624, 4,930)**	**0.65 (0.53, 0.75)**
Greece	3,914,596	733 (517, 938)	159 (111, 202)	543 (376, 705)	0.75 (0.57, 0.88)
Cyprus	480,493	42 (27, 56)	120 (75, 158)	31 (2, 49)	0.78 (0.04, 0.99)
Croatia	1,025,361	523 (419, 616)	461 (370, 543)	332 (225, 441)	0.64 (0.45, 0.80)
Slovenia	334,074	144 (111, 177)	394 (306, 487)	90 (59, 121)	0.63 (0.44, 0.77)
Italy	17,142,001	13,303 (11,829, 14,965)	561 (499, 634)	8,772 (6,239, 10,967)	0.66 (0.48, 0.79)
Malta	200,052	149 (111, 181)	716 (535, 874)	45 (-110, 139)	0.31 (-0.72, 0.88)
Spain	20,054,600	6,824 (5,744, 7,984)	289 (243, 338)	5,227 (3,927, 6,429)	0.77 (0.61, 0.87)
Portugal	3,626,202	476 (383, 606)	112 (90, 142)	243 (157, 342)	0.52 (0.35, 0.66)
**Southern Europe**	**46,777,379**	**22,204 (19,584, 25,086)**	**380 (334, 430)**	**15,255 (11,458, 18,719)**	**0.69 (0.53, 0.80)**
**Europe**	**172,717,680**	**50,391 (46,026, 53,878)**	**276 (252, 295)**	**32,619 (26,078, 38,237)**	**0.65 (0.53, 0.73)**

a Population per country.

b Modelled number of heat-related excess deaths in the factual scenario.

c Standardized excess deaths per million population under the factual scenario.

d Absolute number of deaths attributable to human-caused warming.

e Proportion of deaths attributable to human-caused warming.

b–e We report the median and 95% eCI from 1,000 samples.

## Data Availability

All the datasets used in this study are open access. Temperature data can be downloaded from ERA5-Land (https://cds.climate.copernicus.eu/datasets/reanalysis-era5-land?tab=overview) and E-OBS (https://cds.climate.copernicus.eu/datasets/insitu-gridded-observations-europe?tab=overview), the annual global mean surface temperature from NASA (https://data.giss.nasa.gov/gistemp/index_v3.html) and the multimodel ensemble of coupled global circulation models from the CMIP6 experiments (https://cds.climate.copernicus.eu/data-sets/projections-cmip6?tab=download). Selected data used in the attribution step are available via Zenodo at https://doi.org/10.5281/ zenodo.22794872 (ref. 24). The exposure–response functions at the 854 cities are available via Zenodo at https://doi.org/10.5281/zenodo.7672107 (ref. 25). Mortality data are from Eurostat (https://ec.europa.eu/eurostat/data/database) and population data are from WorldPop (https://www.worldpop.org/).

## Code availability

Code used in the attribution step is available via Zenodo at https://zenodo.org/records/22794872 (ref. 24). Code for performing the health impact assessment is available via GitHub at https://github.com/PierreMasselot/Paper--2023--LancetPH--EUcityTRM.
